# RSF1 in cancer: interactions and functions

**DOI:** 10.1186/s12935-021-02012-9

**Published:** 2021-06-19

**Authors:** Guiyang Cai, Qing Yang, Wei Sun

**Affiliations:** 1 Department of Obstetrics and Gynecology, Shengjing Hospital of China Medical University, Shenyang, China; 2 Department of Key Laboratory of Cell Biology, Ministry of Public Health and Key Laboratory of Medical Cell Biology, School of Life Sciences, China Medical University, Shenyang, China

**Keywords:** RSF1, H2Aub, NF-κB, CENP-A, PLK1, SNF2H

## Abstract

RSF1, remodelling and spacing factor 1, is an important interphase centromere protein and is overexpressed in many types of cancers and correlated with poor overall survival. RSF1 has functions mainly in maintaining chromosome stability, facilitating DNA repair, maintaining the protein homeostasis of RSF1 and suppressing the transcription of some oncogenes when RSF1 protein is expressed at an optimal level; however, RSF1 overexpression facilitates drug resistance and cell cycle checkpoint inhibition to prompt cancer proliferation and survival. The RSF1 expression level and gene background are crucial for RSF1 functions, which may explain why RSF1 has different functions in different cancer types. This review summarizes the functional domains of RSF1**,** the overexpression status of RSF1 and SNF2H in cancer based on the TCGA and GTE_X_ databases, the cancer-related functions of RSF1 in interacting with H2Aub, HDAC1, CENP-A, PLK1, ATM, CENP-S, SNF2H, HB_X_, BubR1, cyclin E1, CBP and NF-κB and the potential clinical value of RSF1, which will lay a theoretical foundation for the structural biology study of RSF1 and application of RSF1 inhibitors, truncated RSF1 proteins and SNF2H inhibitors in the treatment of RSF1-overexpressing tumours.

## Background

The basal packaging unit of the eukaryotic genome is the nucleosome, which is composed of 147 bp of double-stranded DNA wrapped around a histone octamer containing two copies of each histone: H2A, H2B, H3 and H4 [1, 2]. The highly positively charged histone octamer binds to DNA through powerful electrostatic interactions, resulting the complete blocking of the DNA helix from its surrounding environment. Consequently, nucleosomes allow the genome to be accessible to proteins and thus actively regulate genomic transaction processes such as DNA transcription, replication, and repair. Eukaryotic cells can locally affect genomic accessibility by destabilizing specific nucleosomes. This alteration can be achieved through post-translational modifications of histones and/or ATP-dependent chromatin remodellers [2, 3]. ATP-dependent chromatin remodellers are multidomain, evolutionarily conserved enzyme-motor complexes that displace nucleosomes along DNA to remodel chromatin structure, namely, to dynamically regulate nucleosome positions during gene activation and gene suppression. Four structurally related and evolutionarily conserved families have been named after their central ATPases: SWI/SNF, INO80, CHD and ISWI [4, 5]. Mammalian ISWI has two ATPase subunits: SMARCA5 (also known as SNF2H) and SMARCA1 (also known as SNF2L). SNF2H forms five remodelling complexes: ACF, CHRAC, NoRC, WICH and RSF [6]. RSF is composed of SNF2H and RSF1 (remodelling and spacer factor 1, also known as p325). RSF1 remodels the chromatin structure and generates regularly spaced nucleosome arrays, which are a component of interphase centromere proteins (CENPs) and are frequently found to be overexpressed and an adverse prognosticator in many types of cancers, including ovarian [7], breast [8], gallbladder [9], oral squamous cell [10], non-small-cell lung (NSCLC) [11], colon [12], nasopharyngeal cancer (NPC) [13], osteosarcoma (OS) [14], prostate cancer [15], bladder cancer (BC) [16], renal cell carcinoma [17], myxofibrosarcoma [18] and cervical cancer [19]. This review comprehensively summarizes the functional domains of RSF1**,** the overexpression status of RSF1 and SNF2H in cancer based on The Cancer Genome Atlas (TCGA) and Genotype-Tissue Expression (GTE_X_) databases, the cancer-related functions of RSF1 in interacting with histone H2AK119 ubiquitination (H2Aub), histone deacetylase 1 (HDAC1), centromere protein A (CENP-A), polo-like kinase 1 (PLK1), ATM, centromere protein S (CENP-S), SNF2H, hepatitis B virus X (HB_X_), budding uninhibited by benzimidazole-related 1 (BubR1), cyclin E1, CREB binding protein (CBP) and nuclear factor-kappa B (NF-κB) and the potential clinical value of RSF1, which will lay a theoretical foundation for the structural biology study of RSF1 and the application of RSF1 inhibitors, truncated RSF1 proteins and SNF2H inhibitors in RSF1-overexpressing tumours.

## Functional domains of RSF1

**Fig. 1:**
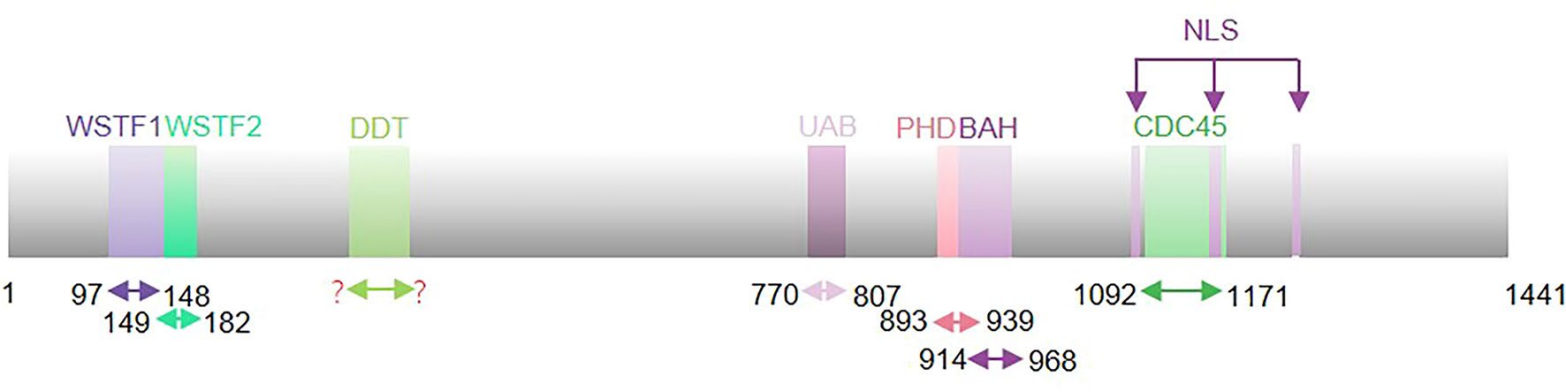
Schematic representation of RSF1 functional domain

RSF1 is a highly acidic protein composed of 1441 amino acids that has many aspartic acids and glutamic acids and a molecular mass of 164 kDa [20]. RSF1 contains two tandem Williams-Beuren syndrome transcription factor (WSTF) domains (aa 97–148 and 149–182), a diphtheria toxin T (DDT) domain [21, 22], a ubiquitinated H2A binding (UAB) domain (aa 770–807), a plant homeodomain-type zinc domain (PHD) (aa 893–939), a bromo adjacent homology (BAH) domain (aa 914–968), a cell division cycle 45 like (CDC45) domain (aa 1092–1171) and three nucleus localization signal sites (NLS) (aa 1084–1091, 1160–1170 and 1237–1244) [20, 22, 23] (Fig. 1). DDT, WSTF1 and WSTF2 have been inferred to comprise an alpha helical module that interacts with nucleosomal linker DNA and the SLIDE domain of ISWI proteins to measure the space between two adjacent nucleosomes [24, 25]. The UAB domain has two segments; the central segment has an α-helical conformation containing two clusters of four conserved aliphatic residues that recognize ubiquitinated proteins, such as the ubiquitin interacting domain, and the N-terminus binds with the nucleosome acidic through an arginine anchoring mechanism [22]. PHD is involved in protein–protein interactions and transcriptional regulation. The BAH domain plays an important role in protein–protein interactions.

## Overexpression status of RSF1 and SNF2H in cancer based on TCGA and GTE_X_ databases

**Fig. 2:**
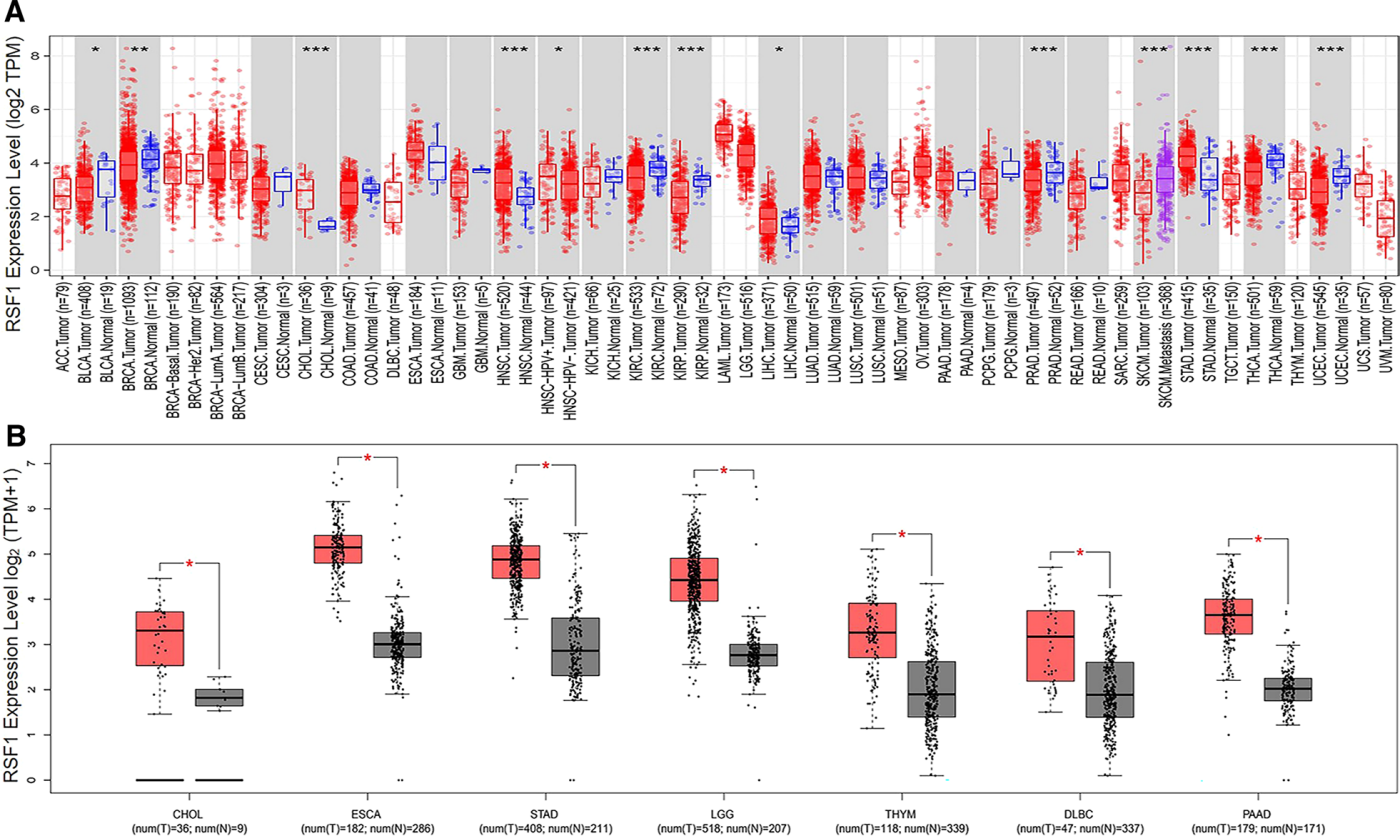
The overexpression status of RSF1 in different tumors compared with the adjacent normal tissues or normal tissues based on TCGA and GTE_X_ databases. TPM represents transcription per million, *P < 0.05, **P < 0.01, ***P < 0.001

**Fig. 3:**
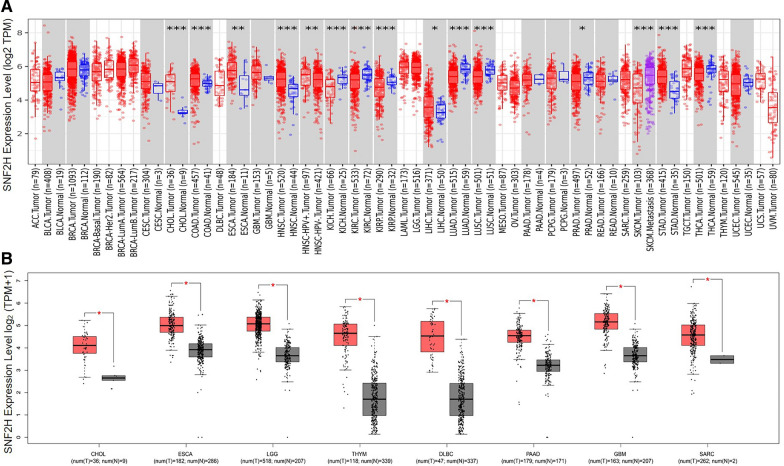
The overexpression status of SNF2H in different tumors compared with the adjacent normal tissues or normal tissues based on TCGA and GTE_X_ databases. TPM represents transcription per million, *P < 0.05, **P < 0.01, ***P < 0.001

We input RSF1 or SNF2H into the “Gene_DE” module of the Tumour Immune Estimation Resource, version 2 (TIMER2) web (http://timer.cistrome.org/) and observed the difference in the expression of RSF1 or SNF2H between tumour and adjacent normal tissues of the TCGA project. We found that RSF1 was overexpressed in cholangiocarcinoma (CHOL), head and neck squamous cell carcinoma (HNSC), liver hepatocellular carcinoma (LIHC) and stomach adenocarcinoma (STAD) tumour tissues compared with adjacent normal tissues (Fig. 2A). SNF2H was overexpressed in CHOL, HNSC, LIHC, STAD, colon adenocarcinoma (COAD), and oesophageal carcinoma (ESCA) tumour tissues compared with adjacent normal tissues (Fig. 3A). For certain tumour datasets without normal tissues [e.g., the adrenocortical carcinoma (TCGA-ACC), basal type breast invasive carcinoma (TCGA-BRCA-Basal), Her2-positive breast invasive carcinoma (TCGA-BRCA-Her2), luminal A type breast invasive carcinoma (TCGA-BRCA-LumA), luminal B type breast invasive carcinoma (TCGA-BRCA-LumB), lymphoid neoplasm—diffuse large B-cell lymphoma (TCGA-DLBC), acute myeloid leukaemia (TCGA-LAML), brain lower grade glioma (TCGA-LGG), mesothelioma (TCGA-MESO), ovarian serous cystadenocarcinoma (TCGA-OV), sarcoma (TCGA-SARC), testicular germ cell tumour (TCGA-TGCT), thymoma (TCGA-THYM), uterine carcinosarcoma (TCGA-UCS), ovarian melanoma (TCGA-UVM), etc. datasets], we used the Gene Expression Profiling Interactive Analysis, version 2 (GEPIA2) web server (http://gepia2.cancer-pku.cn/#analysis) [26] to obtain box plots of the RSF1 or SNF2H expression difference between these tumour tissues and the corresponding normal tissues of the TCGA and GTEx databases under the settings of *P*-value cut-off = 0.05, log2 fold change (log2FC) cut-off = 1, and “match TCGA normal and GTEx data”. We found that RSF1 is overexpressed in CHOL, ESCA, STAD, LGG, THYM, DLBC, and pancreatic adenocarcinoma (PAAD) tumour tissues compared with the corresponding normal tissues (Fig. 2B). SNF2H is overexpressed in CHOL, ESCA, LGG, THYM, DLBC, PAAD, glioblastoma multiforme (GBM), and SARC tumour tissues compared with the corresponding normal tissues (Fig. 3B). It is worth noting that SNF2H is also overexpressed in RSF1-overexpressing tumours based on the TCGA and GTEx databases (Fig. 2 and Fig. 3).

## RSF1 interacts with H2Aub to suppress oncogene transcription

**Fig. 4:**
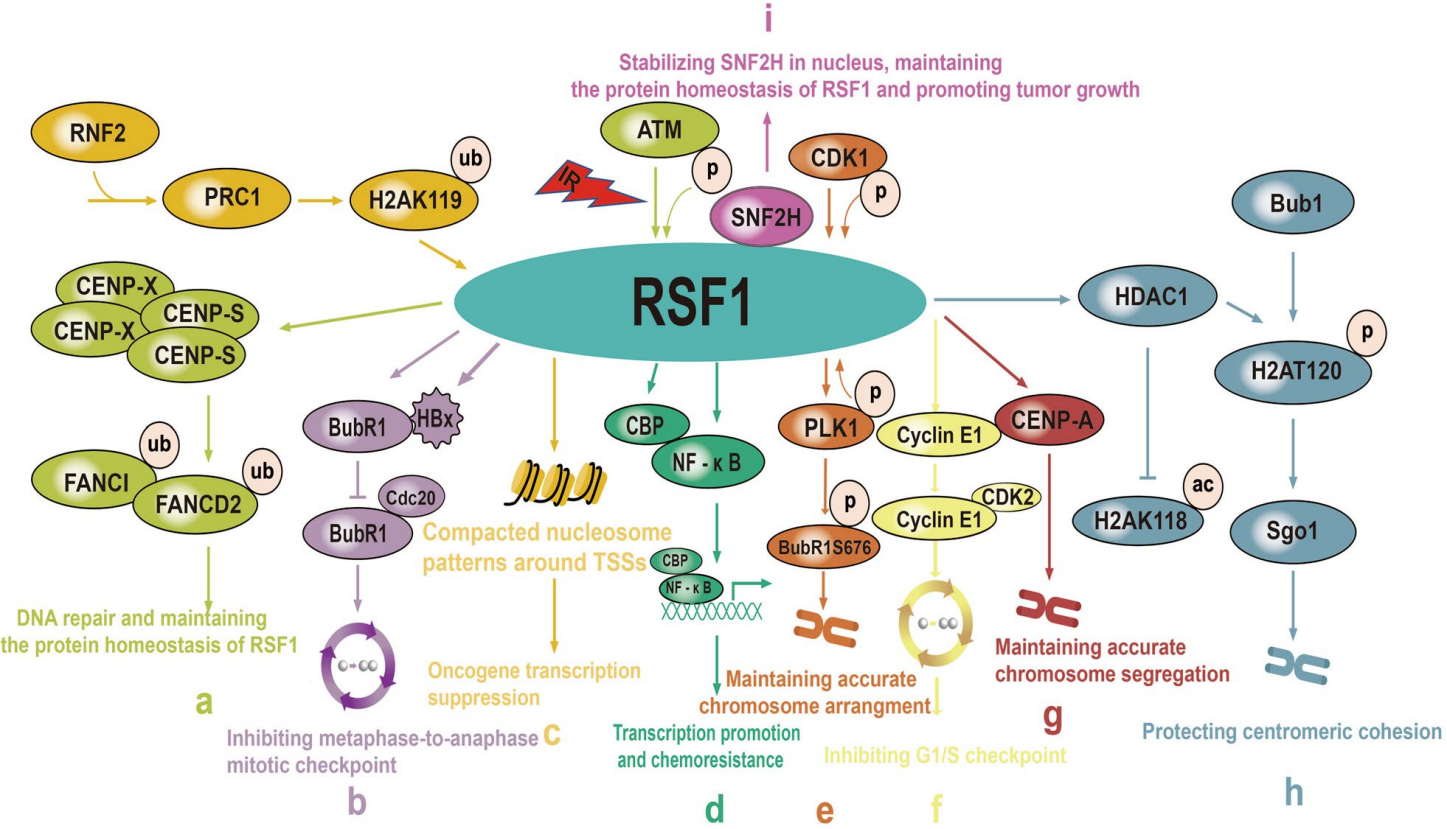
RSF1 interactions and functions involved in cancer. **a** RSF1 interacts with ATM and is phosphorylated by ATM, then to interact with CENP-S/MHF1 to recruit FANCI/FANCD2 at DSBs to promote DNA HR repair, RSF1 interacts with ATM and is phosphorylated by ATM to decrease the upregulated protein level of RSF1 upon DNA damage. **b** RSF1 interacts with HB_X_ and BubR1 to recruit HBX at kinetochore to decrease BubR1-Cdc20 interaction to inhibit metaphase-to-anaphase mitotic checkpoint. **c** RSF1 interacts with H2Aub to suppress oncogene transcription. **d** over-expressed RSF1 recruits CBP to interact with NF-κB, and then activate NF-κB induced transcription to increase chemoresistance. **e** RSF1 interacts with and recruits PLK1 to kinetochore to phosphorylate BubR1 to maintain accurate chromosome arrangement. **f** RSF1 interacts with cyclin E1 to increase CDK2 activity to inhibit G1/S checkpoint. **g** RSF1 recruits CENP-A to centromere to maintain accurate chromosome segregation. **h** RSF1 interacts with and recruits HDAC1 to centromere to protect centromeric cohesion. **i** RSF1 interacts with SNF2H to make SNF2H enter nucleus to stabilize SNF2H, RSF1 interacts with SNF2H to maintain the protein homeostasis of RSF1 in the absence of DNA damage, over-expressed RSF1 interacts with SNF2H to promote tumor growth

Polycomb protein complex 1 (PRC1) subunit ring finger protein 2 (RNF2) acts as the ubiquitin ligase for H2AK119 in humans. PRC1-mediated H2Aub is closely associated with gene silencing, but the mechanism is still obscure. A recent study reported that RSF1 is an H2Aub-binding protein that interacts with H2Aub through a previously uncharacterized UAB domain. The UAB domain specifically recognizes the H2Aub nucleosome through two potential functional fragments not through nonspecific electrostatic binding as previously suspected. RSF1 interacts with H2Aub nucleosomes to organize stable compacted nucleosome patterns around transcription start sites (TSSs) to mediate H2Aub-related gene silencing (Fig. 4c). Although it is unclear why the UAB domain of RSF1 specifically recognizes H2Aub but not H2Bub, it is interesting that the N- and central portions of UAB likely interact with H2Aub additively or synergistically. RSF1 knockout (KO) in HA-ubiquitin-overexpressing HeLa cells results in the dissociation of linker histone H1 from H2Aub nucleosomes. Thus, it remains to be determined how RSF1 or the RSF1-SNF2H complex remodels the H2Aub chromatin conformation to establish stable nucleosome arrays, leading to gene silencing in coordination with linker histone H1 [22].

Chromatin immunoprecipitation and whole genome sequencing (ChIP-seq) found that 82% of H2Aub sites are bound by RSF1, while only 21% of the binding sites of RSF1 are marked with H2Aub [22], which implies that RSF1 may have any other functions on chromatin independent of H2Aub. RSF1 regulates H2Aub-mediated gene silencing, including the classical PRC1 target genes *HOXB8*, *HOXB7* and *HOXC6* [22]. The increased expression of *HOXB8* is associated with colorectal cancer [27], gastric cancer [28], pancreatic cancer [29] and GBM [30]. Increased expression of *HOXB7* is associated with colorectal cancer [31], gastric cancer [32] and prostate cancer [33]. Increased expression of *HOXC6* is associated with gastric cancer [34], laryngeal cancer [35], GBM [36], prostate cancer [37] and oesophageal squamous cell carcinoma [38]. All the above results indicate that RSF1-H2Aub-mediated gene silencing is beneficial for suppressing oncogene transcription. Further study revealed that RNF2-KD, RSF1-KD or SNF2H-KD resulted in upregulated expression of the *SPP1*, *DKK1*, *KCNMA1*, *FBXO2*, *SOCS1* and *KLF2* genes. These data revealed that RSF1 may work together with SNF2H in H2Aub-mediated gene silencing [22]. High *SPP1* expression is associated with recurrence in tamoxifen-treated breast cancer [39]. High *DKK1* expression is associated with bile acid-induced gastric intestinal metaplasia, which is an important precancerous lesion [40]. High *KCNMA1* expression is observed in cervical cancer [41]. High expression of *FBXO2* indicates a high risk of gastric metastasis [42]. High expression of *SOCS1* reverses the inhibitory effect of human papillomavirus (HPV) 16 E1-E2-mediated DNA replication, which is an important cause of cervical cancer [43]. High expression of *KLF2* is related to prostate cancer cell proliferation [44]. All the above, these data further support that RSF1-H2Aub-mediated gene silencing is truly associated with the suppression of oncogene transcription.

## RSF1 interacts with and recruits HDAC1 to centromeres to protect centromeric cohesion

The location of shugoshin 1 (Sgo1) at the centromere can support the maintenance of centromeric cohesion, which requires histone H2A phosphorylation by kinase budding uninhibited by benzimidazole 1 (Bub1) at T120 (H2A-pT120) during early mitosis. RSF1 knockdown (KD) or SNF2H KD causes premature sister chromatid separation (PSCS). RSF1-KD or RSF1-KO cells exhibit impaired Sgo1 localization to centromeres, but this is not due to changes in Sgo1 protein levels. In RSF1-KO cells, H2A-pT120 diffuses from the centromeres throughout the chromosome arms or undetectable, and centromeric accumulation of H2A-K118ac is accompanied by loss of H2A-pT120 at centromeres, which implies that RSF1 recruits Sgo1 to the centromere by maintaining H2A-pT120 at the centromere. HDAC1-deleted cells exhibit a high level of H2A-K118ac in the centrosome region, while the levels of H2A-pT120 and Sgo1 are significantly decreased in the centrosome region. Tandem affinity purification followed by mass spectrometry showed that HDAC1 is an RSF1 binding protein. RSF1 KD significantly reduced the localization of HDAC1 to centromeres and induced an increase in chromatin-bound HDAC1 levels that was not due to changes in HDAC1 protein levels, implying that RSF1 interacts with and recruits HDAC1 to centromeres to maintain H2A-pT120 localization instead of H2A-K118ac localization in the centrosome region. Pull-down analysis revealed that the RSF1-HDAC1 interaction is maintained in asynchronously growing cells (mainly interphase). The C-terminal region (aa 982–1441) of RSF1 is the binding domain of HDAC1. Co-immunoprecipitation showed that the C-terminal LXCXE motif (aa 1244–1248) of RSF1 mutated to 5A lost most of the ability to bind HDAC1. Centromeric HDAC1, H2A-pT120 and Sog1 localization was restored in RSF1-KO cells expressing the C-terminal region (aa 627–1441) or C-terminal region (aa 982–1441). All the results imply that the C-terminal region (aa 627–1441) or C-terminal region (aa 982–1441) of RSF1 can interact with and recruit HDAC1 to centromeres to maintain centromere cohesion [45] (Fig. 3h).

## RSF1 recruits CENP-A to centromeres to maintain accurate chromosome segregation

Centromere, which contains a special nucleosome CENP-A histone, provides not only the basis for centromere chromatin and kinetochore assembly but also a locus for kinetochore-microtubule attachment and spindle assembly checkpoints. Recent studies have emphatically pointed out that ectopic localization of CENP-A induces kinetochore defects and the chromosomal instability phenotype in many cancers [46, 47]. Previously, Obuse C et al. identified that RSF1 and SNF2H co-exist in CENP-A affinity eluates using chromatin immunoprecipitation (ChIP) and mass spectrometric analysis in HeLa cells [48]. Using ChIP and immunofluorescence, Perpelescu M et al. found that CENP-A transiently associates with RSF1 chromatin and localizes at the centromere region starting in early mid-G1 and then dissociates from the centromere, while the input CENP-A amount relative to histone H4 only slightly changes between 1 and 1.6 from G1 to S phase. RSF1/SNF2H depletion in HeLa cells led to the accumulation of 40–60% of cells in prometaphase compared with 30% in the control group; the proportion of misaligned metaphase cells also increased in RSF1-depleted (24%) and SNF2H-depleted (22%) cells compared with control cells (5%), which implies that both RSF1 and SNF2H are necessary for normal mitotic progression. RSF1 depletion rarely reduces cytoplasmic and nuclear CENP-A expression, but a significant decrease in CENP-A was observed in the core chromatin of RSF1-depleted cells, which implies that RSF1 is a newly found factor that maintains CENP-A localization at the centromeric core chromatin [49]. However, we do not know whether RSF1 interacts with CENP-A directly or indirectly via other interphase–centromere complex (ICEN) components or other factors. We also wondered which factors induce RSF1 association with CENP-A chromatin in mid-G1 and which factors induce RSF1 disassociation from CENP-A chromatin. The activity of cyclin-dependent kinases 1 and 2 (CDK1 and CDK2) negatively regulates CENP-A deposition at the centromere, limiting the progression to G1 [50]. CDK1 and CDK2 may induce RSF1 transient interaction with CENP-A at the centromere in G1 by regulating the post-translational modification of RSF1, but this hypothesis needs further study (Fig. 2g).

CENP-A has been reported to localize to laser-induced double-strand damage sites (DSBs), which may be involved in DNA repair [51]. Unfortunately, Pessina F and his colleagues found that RSF1 interacts with CENP-A independent of ionizing radiation (IR) and that CENP-A localization to sites of IR-induced foci was not detected by immunofluorescence. However, RSF1, CENP-S/MHF1 and CENP-A have been detected in ATM immunoprecipitates in U2OS cells treated with IR prepared with formaldehyde (a protein crosslinking reagent) [52]. Therefore, future studies will focus on whether RSF1 works together with CENP-A to perform DNA repair.

## RSF1 interacts with and recruits PLK1 to the kinetochore to phosphorylate BubR1 to maintain accurate chromosome arrangement

PLK1 is an essential mitotic kinase that controls centrosome maturation and maintenance, microtubule attachment to kinetochores and cytokinesis [53]. The accumulation of PLK1 at the kinetochore is necessary for chromosome arrangement [54]. Using immunofluorescence, Ho-Soo Lee et al. showed that RSF1 colocalizes with the inner kinetochore marker ACA at mitotic kinetochores in prometaphase-arrested HeLa and epithelial RPE1 cells. RSF1 depletion results in chromosomal arrangement defects, which are also observed in PLK1-depleted cells. Chromatin fractionation assays also showed that PLK1 at chromatin is reduced in RSF1-depleted HeLa cells, but this is not due to lower overall PLK1 protein levels. These results indicate that RSF1 recruits PLK1 to the kinetochore to maintain accurate chromosome arrangement. Co-immunoprecipitation in vivo and in vitro binding assays showed that RSF1 interacts with PLK1 in mitotic cells, implying that SNF2H is dispensable for the RSF1-PLK1 interaction. The RSF1 protein level is also reduced in the absence of SNF2H [20], but whether SNF2H depletion influences the binding of RSF1 with PLK1 at mitotic kinetochores in HeLa mitotic cells remains to be further studied. A pull-down assay identified that the C-terminal fragment (aa 982–1441) of RSF1 can interact with the C-terminus (aa 350–603) of PLK1, which contains two Polo-box domains (PBDs). One kinase-dead mutant of PLK1 (PLK1 K82R) retained the interaction with RSF1, while other kinase-dead mutants of PLK1 (PLK1 W414F, PLK1 H538A, PLK1 K540M) exhibit reduced interaction with RSF1. All of the above results indicate that these phosphorylation sites in the PBD of PLK1 are indispensable for the RSF1-PLK1 interaction, but we still do not know what factors can cause these phosphorylation sites on PLK1 to change. Further study found that CDK1 phosphorylates RSF1 at Ser1375, which is necessary for PLK1 binding, and then PLK1 phosphorylates RSF1 at Ser1359 to stabilize PLK1 deposition at the mitotic kinetochore (Fig. 2e). Since the common target site of CDK1 is pT/pS-P-X-R/K, another five CDK1 consensus target sites were also found in RSF1, but we still do not know what kind of functions these five CDK1 phosphorylation sites will regulate. However, whether PLK1 phosphorylates other sites of RSF1 and the effects of these phosphorylation sites on the function of RSF1 need to be further studied. RSF1 depletion reduced the level of BubR1 (Ser676), which is specifically phosphorylated by PLK1. As BubR1 phosphorylation is important for the stability of kinetochore–microtubule interactions, these findings may further explain why RSF1 deletion can increase chromosomal arrangement defects. Double knockdown of RSF1 and inner-centromere protein (INCENP) caused a further reduction in chromatin-bound PLK1 levels, implying that RSF1 cooperates with INCENP to stabilize PLK1 [55]. As PLK1 facilitates CENP-A deposition at the centromere through binding with the Mis18 complex [56] and RSF1 is involved in CENP-A deposition at the centromere in G1 phase [49], whether RSF1 cooperates with PLK1 in CENP-A deposition at the centromere needs further study.

## RSF1 interacts with ATM and is phosphorylated by ATM and then interacts with CENP-S/MHF1 to recruit FANCI/FANCD2 at DSBs to promote DNA homologous recombination (HR) repair

RSF1 has been reported to repair IR-induced DNA damage by promoting the aggregation of CENP-S, CENP-X and XRCC4 at DSBs through non-homologous end-joining (NHEJ), and RSF1 recruits CENP-S and CENP-X at sites of DNA damage independent of SNF2H [57]. However, whether RSF1 interacts with CENP-S during DNA NHEJ-mediated repair is not yet known. Another report found that RSF1 is also involved in DNA HR-mediated repair as it recruits the resection factors RpA32 and Rad51, but whether RSF1 recruits RpA32 and Rad51 independent of SNF2H has not been reported. Min S et al. reported that RSF1 and SNF2H begin to aggregate at DSBs 1 min after micro-irradiation and continue to aggregate at DSBs over 30 min after micro-irradiation. Interestingly, RSF1 even accumulates in DSBs continuously within 2 h after micro-irradiation, but the SNF2H signal gradually decreases from DSBs. Depletion of either factor does not affect their mutual accumulation at DSBs [58]. This suggests that RSF1 plays a different role in DNA repair than SNF2H. Further study found that 3 putative motifs (S524, S1226, and S1325) of RSF1 phosphorylated by ATM are important for RSF1 accumulation at DSBs; however, there is a possibility that RSF1 may have more ATM phosphorylation sites in addition to these sites in response to DNA damage because RSF1 has more than 10 ATM phosphorylation sites under DNA damage conditions [58]. Fabio Pessina et al. used co-immunoprecipitation and mass spectrometry and further identified that the ATM interaction with RSF1 is dependent on IR and ATM kinase activity and that active ATM also interacts with SNF2H; however, we still do not know whether the ATM-RSF1 interaction is affected by SNF2H. Then, this group also found that RSF1 directly interacts with CENP-S/MHF1 in IR-treated cells and recruits the CENPS/MHF1-CENPX/MHF2 complex in an orderly manner, which leads to the recruitment and mono-ubiquitination of FANCI/FANCD2 at DSBs for DNA repair [52] (Fig. 2a). As CENP-S/MHF1 is a histone fold protein that most resembles histone H3 protein and RSF preferentially recognizes histone H3 and H3-like CENP-A [49, 59], we speculate that RSF1 may interact with other H3-like proteins. Interestingly, a pull-down assay identified that the C-terminus of RSF1 (aa 1068–1440), which contains the ATM phosphorylation sites (S1226 and S1325), is just the binding domain of CENPS/MHF1, which suggests that RSF1 phosphorylation by ATM is crucial for RSF1-mediated recruitment of CENP-S/MHF1 [52]. Although the precise role of activated FANCI/FANCD2 in DNA repair is not clear, FANCI/FANCD2 localizes at DSBs during the S phase, where HR usually occurs, so we can infer that RSF1/CENP-S/CENP-X/FANCI/FANCD2 may have some role in DNA HR repair [52].

## RSF1 interacts with ATM and is phosphorylated by ATM to attenuate the upregulation of RSF1 protein expression upon DNA damage

RSF1 depletion and overexpression impair DSB repair, indicating that maintaining the protein homeostasis of RSF1 is important for DSB repair signal transduction [60]. The protein level of RSF1 increases temporarily when cells are treated with DNA damage agents, which suggests that attenuating the upregulation of RSF1 protein expression upon DNA damage could enhance the DNA repair function of RSF1. The 3SA mutant (S524A, S1226A, and S1325A) of RSF1 shows a high protein level of RSF1 upon DNA damage because the 3SA mutant is unable to be phosphorylated by ATM [60], which further implies that ATM can attenuate the upregulation of RSF1 protein expression upon treatment with DNA damage agents to maintain the protein homeostasis of RSF1 by phosphorylating RSF1. A previous report found that RSF1 can interact with and is phosphorylated by ATM to interact with CENPS/MHF1 to recruit FANCI/FANCD2 at DSBs to promote DNA HR repair. We speculate that RSF1 interacts with ATM and is phosphorylated by ATM to maintain the protein homeostasis of RSF1 upon DNA damage, which enhances the DNA HR repair effect of RSF1 (Fig. 2a).

## RSF1 interacts with SNF2H to maintain the protein homeostasis of RSF1 in the absence of DNA damage

The RSF1 protein level has been found to decrease rapidly without SNF2H in both the absence and presence of DNA damage [60], which infers that SNF2H is important for maintaining the protein level of RSF1 regardless of DNA damage. Since RSF1 depletion impairs DNA repair, we speculate that SNF2H maintains the protein homeostasis of RSF1 in the absence of DNA damage, which is conducive to RSF1 DNA repair. As the protein homeostasis of RSF1 is so important for its DNA repair function [60], further studies need determine which other factors can maintain RSF1 protein homeostasis (Fig. 2i).

## RSF1 interacts with SNF2H to facilitate SNF2H nuclear entry to stabilize SNF2H

Sheu JJ et al. confirmed for the first time that RSF1 and SNF2H are co-upregulated in high-grade ovarian serous carcinoma tissues by immunohistochemistry, and both of proteins are expressed in the nucleus. The interaction of RSF1 with SNF2H in OVCAR3 cells with endogenous RSF1 overexpression was identified by co-immunoprecipitation. The expression of RSF1 was induced by the Tet-off system in SKOV3 cells without RSF1 amplification and RSF1 expression, and the SNF2H protein level increased in a time-dependent manner as RSF1 expression increased. In addition, the mRNA level of SNF2H increased only 1.04-fold after 6 h of RSF1 induction, suggesting that the RSF1 protein may have a stabilizing effect on SNF2H protein levels in cancer cells. In the RSF1-induced RK3E cell line, the SNF2H protein is widely distributed in the cytoplasm and nucleus when RSF1 is turned off, but when RSF1 is turned on, SNF2H translocates to the nucleus and co-localizes with RSF1, indicating that RSF1 interacts with SNF2H and recruits SNF2H to the nucleus [21] (Fig. 2i).

## Overexpressed RSF1 interacts with SNF2H to promote tumour growth

Induced expression of RSF1 promoted the growth of SKOV3 xenografts compared with that of non-induced cell xenografts; RSF1 (aa 1–973) but not the RSF1 (aa 1–441) is the only truncated protein that coimmunoprecipitates with SNF2H (and thus regulates the RSF1-SNF2H interaction), so RSF1 (aa 1–973) truncated protein can significantly inhibit the growth of ovarian cancer cells with RSF1 gene amplification and overexpression [21] (Fig. 2i).

## Overexpressed RSF1 recruits SNF2H from other remodelling complexes to the RSF remodelling complex

Sheu JJ et al. also found that compared with that in non-induced SKOV3 cells, the amount of BAZ1A and BAZ1B coprecipitated with SNF2H in SKOV3 cells with RSF1-induced expression was significantly reduced. Compared with OVCAR3 cells without RSF1 knockdown, the amount of BAZ1A and BAZ1B co-precipitated by SNF2H was significantly increased in OVCAR3 cells with RSF1 knockdown. Because BAZ1A, BAZ1B and BAZ2A form other remodelling complexes with SNF2H, RSF1 recruits SNF2H from other remodelling complexes to the RSF remodelling complex in RSF1-overexpressing tumours [21]. As chromatin remodelling complexes are closely associated with the development and differentiation of cells, SNF2H recruited from other remodelling complexes to the RSF remodelling complex by overexpressed RSF1 could have a notable effect on the biological function of cancer cells (Fig. 2i).

## RSF1 interacts with HB_X_ and BubR1 to recruit HB_X_ to the kinetochore to decrease the BubR1-Cdc20 interaction to inhibit the metaphase-to-anaphase mitotic checkpoint

LIHC is a major malignant and lethal tumour that is the most common in Asian patients with chronic hepatitis B virus (HBV) infection [61, 62]. The HBV genome encodes DNA polymerase, surface antigen, core protein and HB_X_ [63]. HB_X_ has been shown to enhance HBV replication and promote HBV-induced hepatocarcinogenesis [64, 65], but the mechanism of HBV-induced hepatocarcinogenesis is still obscure. Previously, Sunyoung Chae et al. found that only full-length RSF1 interacts with both HB_X_ and BubR1 in mitotic HeLa cells. Depletion of RSF1 does not influence the localization of BubR1 to the kinetochore but does dramatically disrupt HB_X_ kinetochore localization. The Kunitz domains (aa 61–76 and 133–145) of HB_X_ interact with the Cdc20 binding domain of BubR1. The interaction between BubR1 and Cdc20 dramatically decreased with increasing HB_X_ concentration. The frequency of defective mitotic events, such as chromosome bridges and lagging chromosomes, in RSF1-depleted cells (30%) was decreased compared with that in cells only transfected with the HB_X_ overexpression plasmid (55%). BubR1 is usually located in the centromeric chromatin region, where it has been shown to bind directly to Cdc20, thereby inhibiting the metaphase-to-anaphase transition by inhibiting the activation of anaphase-promoting complex/cyclosome (APC/C) [66]. All the above results suggest that RSF1 recruits HB_X_ to kinetochores to potentiate the HB_X_-BubR1 interaction and weaken the BubR1-Cdc20 interaction to decrease the metaphase-to-anaphase mitotic checkpoint to increase chromosome instability (Fig. 2b). A recent study also reported that the region inhibited by gene-targeted therapy encodes a conserved amino acid region (aa 63–76) of HB_X_ that partially overlaps with a Kunitz domain of HB_X_, which is of value in HBV-associated hepatocellular carcinoma therapy regardless of the clinical stage or HBV genotype of the patient [67]. Thus, RSF1 inhibitors can be used to reduce the incidence of HBV-associated hepatocellular carcinoma by preventing RSF1 from recruiting HB_X_ to kinetochores in RSF1-overexpressing LIHC.

## RSF1 interacts with cyclin E1 to increase CDK2 activity to inhibit the G1/S checkpoint

Whole-genome digital karyotype analysis has shown that the *RSF1* and *CCNE1* genes are co-amplified in OVCAR3 cells; RSF1 and cyclin E1 are co-upregulated in high-grade serous ovarian carcinoma, and a high prevalence of *TP53*^*mut*^ (> 85%) also exists in high-grade serous ovarian carcinoma [68, 69]. Co-immunoprecipitation followed by mass spectrometry and peptide sequencing confirmed that cyclin E1 is one of the main direct interacting proteins of RSF1 in RSF1-overexpressing OVCAR3 cells. SNF2H can only bind with cyclin E1 in RSF1 expression-induced SKOV3 ovarian cancer cells, implying that cyclin E1 binds with SNF2H through direct binding with RSF1. Ectopic expression of RSF1 together with cyclin E1 and human p53^R645H^ in RK3E cells leads to cell proliferation and invasion and abnormal mitotic figures (tripolar or tetrapolar metaphase). If only cyclin E1 or RSF1 was expressed or both were co-expressed on the *TP53*^*wt*^ background, tumorigenesis was not detected. All these results show that RSF1 interacts with cyclin E1 to promote tumorigenesis only on the *TP53*^*mut*^ background. Cyclin E1 forms a complex with CDK2 to activate CDK2, which is necessary for the G1/S transition [70]. Kinase activity assays confirmed that high levels of RSF1 and cyclin E1 can enhance CDK2 activity, indicating that RSF1 inhibits the G1/S checkpoint by interacting with cyclin E1 to activate CDK2. Although a pull-down assay confirmed that cyclin E1 binds with the first 441 amino acids of RSF1, full-length RSF1, but not the minimal binding domain of RSF1, has a tumour-promoting function. The truncated RSF1 protein (aa 1–441) reduces tumorigenicity in mice with a *TP53*^*mut*^ background suggesting that this truncated protein of RSF1 can be used as a therapeutic drug in *TP53*^*mut*^ cancers to compete with RSF1 for interacting with cyclin E1 to inhibit the cancer-promoting functions of RSF1 [23] (Fig. 2f).

## Overexpressed RSF1 in KRAS mutation-driven cancers bypasses the Gln deprivation-induced G1 checkpoint to decrease chemoresistance

Cancer cells with KRAS mutations bypass the Gln deprivation-induced G1 checkpoint and instead are blocked in S phase. KRAS mutation-driven tumours stagnate in S phase due to a lack of aspartic acid and are prone to apoptosis induced by the cytotoxic drugs capecitabine, paclitaxel, and rapamycin [71–73]. However, the G1 arrest induced by Gln deprivation can be recovered by inhibiting ERK and mTOR, which are downstream effectors of KRAS. As RSF1 is overexpressed in both OS, NSCLC and PAAD, carcinogenic KRAS mutations are present in approximately 30% of human cancers and more than 90% of PAAD, RSF1 inhibition in OS and NSCLC cells inactivates the ERK signalling pathway [11, 14], which suggests that KRAS mutation-driven cancers with high protein levels of RSF1 increase sensitivity to cytotoxic drugs.

## Overexpressed RSF1 recruits CBP to interact with NF-κB and then activates NF-κB-induced transcription to increase chemoresistance

The NF-κB family includes precursor molecules (p105 and p100) and other proteins (p50 and p52), which are acquired by proteolysis of the two precursors, which removes their C-terminal ankyrin repeats. p50 and p52 can regulate transcription only when they form heterodimers with other NF-κB factors (p65, RelB and c-Rel) that contain a transactivation domain [74, 75]. The activation of NF-κB is associated with chemoresistance in gastric cancer [76], colorectal cancer [77], gallbladder cancer [78], NSCLC [79], ovarian cancer [80] and pancreatic cancer [81]. Although DNA damage-induced NF-κB activation is the main reason for chemoresistance [82], it is unclear whether other factors can cause the activation of NF-κB to promote chemoresistance in the abovementioned cancers.

Recently, RSF1 gene amplification was found in ovarian cancer [7]. RSF1 is upregulated in paclitaxel-resistant ovarian cell lines and is significantly associated with paclitaxel resistance. SNF2H downregulation or disruption of the interaction between SNF2H and RSF1 enhances paclitaxel sensitivity in tumour cells with RSF1 overexpression [83]. RSF1 is a transcriptional activator of NF-κB-dependent gene transcription and increases NF-κB/P65 protein expression without changes in NF-κB mRNA expression. Further co-immunoprecipitation assays identified increased RSF1 binding with NF-κB in RSF1 expression-induced SKOV3 ovarian cancer cells; an interaction between RSF1 and NF-κB was also observed in endogenous RSF1-overexpressing OVCAR3 cells and RSF1-overexpressing SKOV3^TR^ and OVCAR3^TR^ cells, which are paclitaxel-resistant ovarian cancer cells. We also found that RSF1 binds with CBP (a ubiquitous coactivator for NF-κB activity) in RSF1 expression-induced SKOV3 and OVCAR3 cells. ChIP assays further identified that RSF1 and CBP bind to the consensus NF-κB element in the prostaglandin-endoperoxide synthase 2 (PTGS2) and X-linked inhibitor of apoptosis (XIAP) promoters of SKOV3^TR^ cells. These data suggest that RSF1, as a bridging factor, recruits CBP to interact with and activate NF-κB to increase *PTGS2* and *XIAP* gene transcription to increase chemoresistance [84]. Rushworth et al. demonstrated that p65 induces nuclear factor (erythroid-derived 2)-like 2 (Nrf2) transcription, which is the prime cause of bortezomib resistance in acute myeloid leukaemia (AML) cells [85, 86]. Nrf2 activation also results in gemcitabine resistance in pancreatic ductal adenocarcinoma cells [87]. All these results indicate that increased RSF1 expression can promote the development of chemoresistance by increasing the transcriptional activity of p65 and then promoting the transcription and activation of Nrf2. A strong correlation between high levels of RSF1 in ovarian cancer and increased expression of NF-κB-targeted genes involved in evasion of apoptosis (CFLAR, XIAP, BCL2, and BCL2L1) and inflammation (PTGS2) has also been found. Further study found that RSF1 overexpression in nasopharyngeal carcinoma CEN-2 cells increases the expression of the NF-κB targeted gene *SURVIVIN*, thereby enhancing paclitaxel resistance by activating NF-κB [88]. All the results suggest that RSF1 inhibitors may attenuate the induction of chemoresistance by inhibiting NF-κB transcriptional activity in various cancers with RSF1 overexpression (Fig. 3d).

## The potential clinical value of RSF1

Clinically, RSF1 expression is increased and is associated with late clinical features and poor overall survival in patients with OS. Decreased expression of miR-193a-3p is associated with advanced clinical features and low overall survival in patients with OS. In addition, miR-193a-3p expression is negatively correlated with RSF1 expression in OS. In conclusion, miR-193a-3p can be used as an RSF1 inhibitor in the treatment of OS [14]. Compared with that in normal tissues and cells, small nucleolar RNA host gene 6 (SNHG6) and RSF1 expression are upregulated, while miR-490-3p expression is downregulated, in NSCLC tumours and cell lines. In addition, SNHG6 promotes the proliferation and inhibits the apoptosis of NSCLC cells by regulating the miR-490-3p/RSF1 axis. Therefore, SNHG6 inhibitors or miR-490-3p can be used in the treatment of NSCLC cancer as they inhibit the expression of RSF1 [89]. Progression-associated lncRNA in breast cancer (PRLB) expression is significantly enhanced in paclitaxel-resistant ovarian cancer tissues and cells. Knockdown of PRLB has been identified at least partly to improve the sensitivity of ovarian cancer cells to paclitaxel by inhibiting miR-150-5p to further inhibit the activation of RSF1/NF-κB signalling. PRLB inhibitors or RSF1 inhibitors must play an important role in increasing the sensitivity of ovarian cancer to paclitaxel [90]. High expression levels of lncRNA nuclear paraspeckle assembly transcript (NEAT1) and RSF1 and low expression levels of miR-1224-5p coexist in gastric cancer. Upregulation of NEAT1 or knockdown of miR-1224-5p prompts gastric cancer cell proliferation and migration. NEAT1 inhibitors and RSF1 inhibitors have opened up new prospects for the treatment of gastric cancer [91]. The expression level of miR-154 in BC is significantly lower than that in adjacent normal tissues. The expression of RSF1 is negatively correlated with miR-154 in BC. MiR-154 significantly inhibits the proliferation, migration and invasion of T24 cells through targeted inhibition of RSF1 expression [16], indicating that RSF1 inhibitors can provide brightness for BC cancer therapy. RSF1 is upregulated in cervical cancer, and RSF1 siRNA combined with radiation can inhibit cell viability, redistribute the cell cycle and induce apoptosis of HeLa and SiHa cell lines. RSF1 inhibitors may be a promising way to develop new radiosensitizers for cervical cancer [19]. Recently, another report pointed out that the lncRNA NEAT1/let-7a-5p axis regulates cisplatin resistance in NPC by targeting RSF1, implying that RSF1 inhibitors and NEAT1 inhibitors can be used to increase the sensitivity of NPC cells to cisplatin [92].

### Conclusions

RSF1 is a protein that tends to interact with H3-like proteins, and the binding domain with which RSF1 interacts with other proteins is not limited to the C-terminus. The N-terminus of RSF1 can also bind with SNF2H and cyclin E1. RSF1 expressed at the optimal level can interact with H2Aub, HDAC1, CENP-A, PLK1, ATM, CENP-S and SNF2H to play an important role in oncogene transcription suppression, protecting centromeric cohesion, maintaining accurate chromosome segregation and arrangement, DNA HR repair, maintaining the protein homeostasis of RSF1 and stabilizing SNF2H in the nucleus, which are good for cancer prevention; however, under high expression conditions, RSF1 interacts with SNF2H, BubR1, HB_X_, cyclin E1, CBP and NF-κB to play a part in promoting tumour growth, recruiting SNF2H from other remodelling complexes to the RSF remodelling complex, inhibiting the metaphase-to-anaphase mitotic checkpoint, promoting tumorigenesis only on the *TP53*^*mut*^ background and activating NF-κB-induced transcription to increase chemoresistance, which support cancer progression and limit the efficacy of cancer drugs (Table 1). Thus, RSF1 inhibitors have good application prospects for cancers with RSF1 overexpression. As both SNF2H and RSF1 are always overexpressed in the same cancer type, SNF2H has the function of maintaining the protein homeostasis of RSF1, and the protein level of SNF2H influences the interaction between RSF1 and other RSF1 binding proteins; thus, SNF2H inhibitors can be used in cancers with RSF1 overexpression, which provides a new treatment strategy for these kinds of cancers. Truncated RSF1 proteins can interfere with the interaction between RSF1 and other proteins and can also be used in cancers with RSF1 overexpression, especially those with a *TP53*^*mut*^ background. In particular, it should be noted that the high protein level of RSF1 is not always an unfavourable factor for cancers. In cancers with KRAS mutations, RSF1 overexpression can increase the sensitivity of KRAS mutation-driven cancers to cytotoxic drugs.

**Table 1:** Function, RSF1 binding domain and SNF2H’s effects on function of different protein level of RSF1 with different RSF1 binding protein

The protein level of RSF1	RSF1 binding protein	Function	RSF1 binding domain	SNF2H’s effects on function
Normal	H2Aub	Oncogene transcription suppression	?	Yes
Normal	HDAC1	Protecting centromeric cohesion	aa 982–1441	Yes
Normal	CENP-A	Maintaining accurate chromosome segregation	?	Yes
Normal	PLK1	Maintaining accurate chromosome arrangment	aa 982–1441	?
Normal	ATM	DNA HR repair	?	?
Normal	CENP-S	DNA HR repair	aa 1068–1440	?
High	ATM	Decreasing the upregulated protein level of RSF1 upon DNA damage	?	?
Normal	SNF2H	Maintaining the protein homeostasis of RSF1	?	Yes
Normal	SNF2H	Stabilizing SNF2H in nucleus	aa 1–973	Yes
High	SNF2H	Promoting tumor growth	aa 1–973	Yes
High	SNF2H	Scrambling SNF2H from other remodeller complexes to RSF remodeller complex	?	Yes
High	BubR1	Inhibiting metaphase-to-anaphase mitotic checkpoint	?	?
High	HB_X_	Inhibiting metaphase-to-anaphase mitotic checkpoint	?	?
High	Cyclin E1	Promoting tumorigenesis only in *TP53*^*mut*^ background	aa 1–441	?
High	CBP	Activating NF-κB induced transcription to increase chemoresistance	?	Yes
High	NF-κB	Activating NF-κB induced transcription to increase chemoresistance	?	Yes

## Data Availability

Not applicable.

## Abbreviations

RSF1: Remodelling and spacing factor 1
CENPs: Interphase centromere proteins
NSCLC: Non-small-cell lung cancer
NPC: Nasopharyngeal cancer
OS: Osteosarcoma
BC: Bladder cancer
H2Aub: Histone H2AK119 ubiquitination
HDAC1: Histone deacetylase 1
CENP-A: Centromere protein A
PLK1: Polo-like kinase 1
CENP-S: Centromere protein S
HB_X_: Hepatitis B virus X
BubR1: Budding uninhibited by benzimidazole-related 1
CBP: CREB binding protein
NF-κB: Nuclear factor-kappa B
WSTF: Williams–Beuren syndrome transcription factor
DDT: Diphtheria toxin T
UAB: Ubiquitinated H2A binding
PHD: Plant homeodomain-type zinc domain
BAH: Bromo adjacent homology
CDC45: Ell division cycle 45 like
NLS: Nucleus localization signal sites
TIMER2: Tumour immune estimation resource, version 2
CHOL: Cholangiocarcinoma
HNSC: Head and neck squamous cell carcinoma
LIHC: Liver hepatocellular carcinoma
STAD: Stomach adenocarcinoma
COAD: Colon adenocarcinoma
ESCA: Oesophageal carcinoma
GEPIA2: Gene Expression Profiling Interactive Analysis, version 2
TCGA: The Cancer Genome Atlas
GTEx: Genotype-Tissue Expression
LGG: Brain lower grade glioma
THYM: Thymoma
DLBC: Lymphoid neoplasm diffuse large B-cell lymphoma
PAAD: Pancreatic adenocarcinoma
GBM: Glioblastoma multiforme
SARC: Sarcoma
PRC1: Polycomb protein complex 1
RNF2: Ring finger protein 2
TSSs: Transcription start sites
ChIP-seq: Chromatin IP and whole genome sequencing
HPV: Human papillomavirus
Sgo1: Shugoshin1
Bub1: Budding uninhibited by benzimidazole 1
PSCS: Premature sister chromatid separation
ChIP: Chromatin immunoprecipitation
ICEN: Interphase–centromere complex
CDK1/CDK2: Cyclin-dependent kinase 1/cyclin-dependent kinase 2
DSBs: Double strand damage sites
PBDs: Polo-box domains
INCENP: Inner-centromere protein
HR: Homologous recombination
NHEJ: Non-homologous end-joining
HBV: Hepatitis B virus
APC/C: Anaphase-promoting complex/cyclosome
CDK2: Cyclin dependent kinase 2
PTGS2: Prostaglandin-endoperoxide synthase 2
XIAP: X-linked inhibitor of apoptosis
Nrf2: Nuclear factor (erythroid-derived 2)-like 2
SNHG6: Small nucleolar RNA host gene 6
PLB: Progression-associated lncRNA in breast cancer
NEAT1: Nuclear paraspeckle assembly transcript
